# Targeted Deletion of Multiple CTCF-Binding Elements in the Human C-MYC Gene Reveals a Requirement for CTCF in C-MYC Expression

**DOI:** 10.1371/journal.pone.0006109

**Published:** 2009-07-01

**Authors:** Wendy M. Gombert, Anton Krumm

**Affiliations:** 1 Black Lowe & Graham PLLC, Seattle, Washington, United States of America; 2 Department of Radiation Oncology, University of Washington School of Medicine, Seattle, Washington, United States of America; 3 Institute for Stem Cell and Regenerative Medicine, University of Washington School of Medicine, Seattle, Washington, United States of America; Roswell Park Cancer Institute, United States of America

## Abstract

**Background:**

Insulators and domain boundaries both shield genes from adjacent enhancers and inhibit intrusion of heterochromatin into transgenes. Previous studies examined the functional mechanism of the MYC insulator element MINE and its CTCF binding sites in the context of transgenes that were randomly inserted into the genome by transfection. However, the contribution of CTCF binding sites to both gene regulation and maintenance of chromatin has not been tested at the endogenous MYC gene.

**Methodology/Principal Findings:**

To determine the impact of CTCF binding on MYC expression, a series of mutant human chromosomal alleles was prepared in homologous recombination-efficient DT40 cells and individually transferred by microcell fusion into murine cells. Functional tests reported here reveal that deletion of CTCF binding elements within the MINE does not impact the capacity of this locus to correctly organize an ‘accessible’ open chromatin domain, suggesting that these sites are not essential for the formation of a competent, transcriptionally active locus. Moreover, deletion of the CTCF site at the MYC P2 promoter reduces transcription but does not affect promoter acetylation or serum-inducible transcription. Importantly, removal of either CTCF site leads to DNA methylation of flanking sequences, thereby contributing to progressive loss of transcriptional activity.

**Conclusions:**

These findings collectively demonstrate that CTCF-binding at the human MYC locus does not repress transcriptional activity but is required for protection from DNA methylation.

## Introduction

Insulator and boundary elements shield genomic loci from unscheduled transcriptional activation or repression by regulatory elements. In higher eukaryotes, the majority of insulator elements bind to the highly conserved zinc-finger protein CTCF which has been shown to mediate diverse regulatory functions including promoter activation and repression, gene silencing, insulation, and imprinting [1], [2].

Recent genome-wide surveys have demonstrated that CTCF is uniquely distributed throughout the genome in a pattern distinct from general transcription factors [3], [4]. These studies have revealed that the majority of CTCF binding sites are located far from gene promoters with only one fifth of CTCF binding sites mapping within 2 kb of transcription initiation sites. Interestingly, promoter-proximal CTCF-binding sites generally correlate with low gene activity [4], consistent with previous reports that CTCF can repress transcription [5], [6].

**Figure 1:**
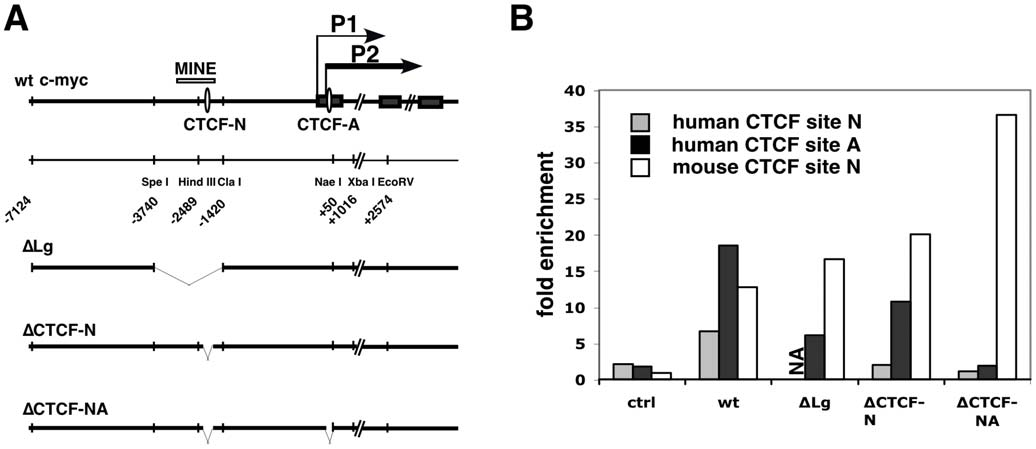
Structure of recombinant human MYC alleles containing deletions in CTCF binding regions. (A) Schematic outline of the wildtype human MYC gene with its three exons (dark filled bars), the MYC insulator element MINE and the positions of CTCF binding site N and A. Black arrows indicate transcriptional start sites of the P1 and P2 MYC promoters. Depicted below are the promoter regions deleted in MYC alleles ΔLg, ΔCTCF-N and ΔCTCF-NA. (B) ChIP analysis of CTCF binding at the wildtype and mutant human and mouse MYC alleles. Bars show the fold enrichment of CTCF binding sequences relative to the enrichment of β-globin sequences. While CTCF binding is detectable at the wildtype (wt) human MYC allele, a 163 bp deletion at site N (ΔCTCF-N) and a 54 bp deletion at site A (ΔCTCF-NA) abrogate binding. In mock immunoprecipitations (ctrl, without antibody), none of the CTCF-binding sequences is enriched. Enrichment of CTCF site N is not analyzed (NA) in the mutant ΔLg as sequences for the primer set used is deleted.

CTCF repressor activity was first described for the MYC gene. The arrangement of CTCF-binding sites within the human MYC gene is one of the notable exceptions to the general genomic pattern outlined above. The mammalian MYC gene is embedded in a gene-poor environment within an extensive region of condensed chromatin, which is bordered on either side by matrix-associated elements. A constitutive CTCF binding site, associated with a DNAse I hypersensitive region, termed MINE (MYC insulator element), is able to act as an insulator in stably transfected cell lines [7]. The CTCF-binding site within the MINE (site N, Figure 1) is coincident with the boundary of hyperacetylation of the MYC promoter. This arrangement suggested that CTCF functions as a barrier for the MYC locus by the formation and/or maintenance of chromatin structure required for normal MYC gene expression. In addition to CTCF site N, an additional constitutive CTCF-binding site (site A, Figure 1) is located close to the transcriptional start site of the MYC P2 promoter. Deletion of site A in a transiently transfected MYC reporter gene construct has been reported to relieve suppression of MYC promoter activity [6].

Although the exact mechanism through which CTCF impacts transcriptional activity remains elusive, previous data suggest that its multiple effects on transcription are attributable to its capacity to mediate the formation of inter- and intra-chromosomal loops [8]. Insulator-mediated formation of chromatin loop structures has been demonstrated in both Drosophila and mammalian cell types (reviewed in [9], [10]). For example, allele-specific binding of CTCF to the imprinting control region of the IGF2/H19 gene locus mediates the allele-specific interaction of promoters with regulatory elements on different chromosomal locations [11], [12]. This allele-specific interaction is thought to activate and silence the paternal and maternal IGF2 allele, respectively.

Conceptually, the multiple roles for CTCF in gene regulation could be due to its ability to mediate chromatin loops [10], [13]. Indeed, the formation of reversible chromatin loops through regulatory proteins can block the activity of the viral SV40 enhancer [14]. While present data suggest an essential role for CTCF in nuclear organization, the majority of its effects on transcription were derived from analyses using CTCF sites outside of their naturally occurring chromatin domain.

Given the potential significance of the CTCF-binding arrangement at the MYC promoter, we have determined the consequences of CTCF binding site deletions at the human MYC gene in the context of the complete human chromosome. These data indicate that CTCF is required for normal MYC expression. Deletion of CTCF sites N and A at this locus results in reduced expression of the MYC gene. Reduced transcription correlates with an increase in levels of DNA methylation within the promoter and around the deleted CTCF binding sites. These data are inconsistent with a role for CTCF as a transcriptional repressor of MYC expression, and support previously proposed models in which CTCF is essential for protecting gene domains from DNA methylation.

## Results

### Deletion of CTCF binding sequences at the MYC gene by homologous recombination

The MYC insulator element MINE serves as an efficient boundary and insulator element for both transfected and randomly integrated reporter-gene constructs [7]. To test whether CTCF is essential for the insulator function of MINE, we engineered targeted deletions within the MYC locus on human chromosome 8 using the recombination-efficient cell line DT40 [15]. In order to examine the consequence of CTCF binding site deletions in mammalian cells, the recombined human chromosomes were subsequently transferred into murine B78 cells by microcell fusion.

We created three different deletions within the MYC insulator and promoter regions (Figure 1A). A large 2.3 kb deletion (ΔLg) removed sequences from 1.4 kb to 3.7 kb upstream of the P2 transcription initiation site, including the entire MINE and additional flanking sequences. To further elucidate the role of CTCF in chromatin insulation at the MYC locus, we generated two smaller deletions ΔCTCF-N and ΔCTCF-NA (163 bp and 54 bp, respectively) that removed CTCF binding sites either within the MINE region or within both the MINE and the MYC promoter (Figure 1A).

To confirm that these deletion alleles eliminate CTCF binding, we performed ChIP analysis using chromatin isolated from B78 cell lines containing wildtype or mutated MYC alleles. Quantitative PCR analyses confirmed that the wildtype human MYC allele –after transfer into murine B78 cells - binds CTCF at both the MINE and the P2 proximal promoter (Figure 1B). In contrast, targeted deletion of CTCF binding sites N and A in human MYC alleles (ΔCTCF-N and ΔCTCF-NA) predictably abolished recruitment of CTCF at these sites. Of note, CTCF-binding to the P2 promoter-proximal site occurs independently of CTCF-binding within the MINE. CTCF site A of the MYC P2 promoter region remains associated with CTCF even after loss of CTCF binding at site N. These data do not preclude the possibility that each of these sites independently recruit CTCF, but act in concert to regulate transcription.

### MYC adopts an active chromatin structure upon transfer into murine B78 cells

**Figure 2:**
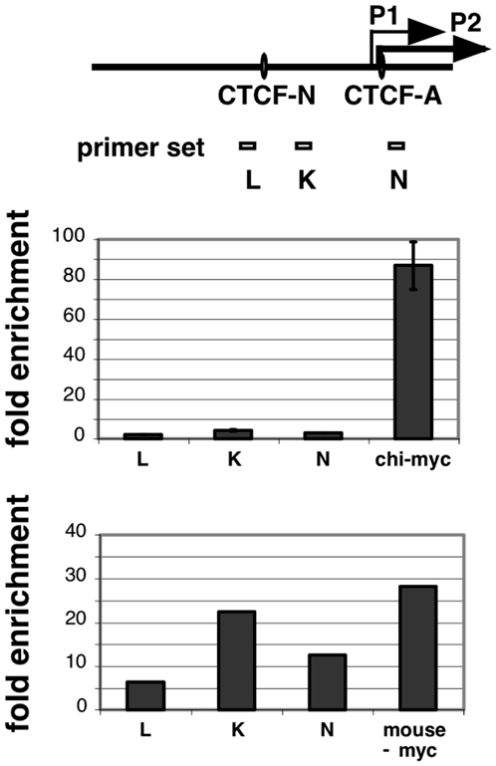
Acetylation status of the MYC locus on human chromosome 8 residing in chicken DT40 cells and in murine B78 cells. DNA recovered from chromatin immunoprecipitations with antibodies specific for lysine9/14 acetylated histone H3 (H3K9/14ac) was subjected to duplex PCR using primers specific for the human, chicken and mouse MYC genes. The enrichment of these regions in the immunoprecipitated DNA was normalized to the signal detected with primers specific for the endogenous chicken β-globin gene or the mouse β-globin. Upper graph, comparison of histone acetylation levels at the human MYC promoter regions L, K, N and the chicken MYC promoter region in DT40 cells. Primer location and CTCF sites N and A of the human MYC promoter region are shown schematically at the top. Lower panel, comparison of histone acetylation levels at human MYC promoter regions L, K, N, and the murine MYC promoter region after transfer of human chromosome 8 into mouse B78 cells.

**Figure 3:**
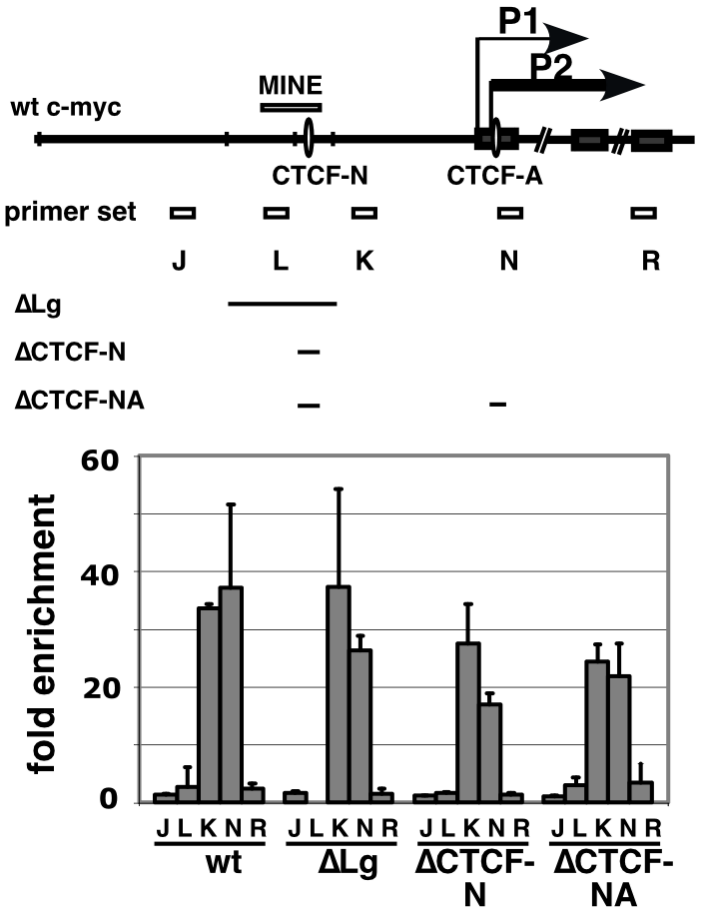
Acetylation status of wildtype and mutated MYC loci on human chromosome 8. ChIP experiments were performed with antibodies specific for H3K9/14ac using chromatin from mouse B78 clones carrying wildtype or mutated MYC loci on human chromosome 8. The binding sites for CTCF within the wildtype MYC promoter, the positions of primers sets used to amplify regions (amplicons) J through R, and the location of deletions introduced into the human MYC promoter are shown above. Arrows indicate transcriptional start sites of the P1 and P2 promoter. The fold enrichment of acetylated histone H3 was normalized to the signals from the endogenous mouse β-globin gene promoter as an internal standard (mean±stdev, n = 3).

To determine whether any of the deleted DNA elements at the boundary of the MYC domain are required for the establishment or maintenance of transcriptionally active or inactive chromatin structures, we compared the degree of histone modification at human MYC alleles relative to the homologous allele residing in chicken DT40 and murine cells. ChIP-analysis of chromatin prepared from DT40 cells containing the human chromosome 8 [DT40(hc8)] revealed that the human MYC promoter displayed a 21-fold lower level of histone H3 acetylation compared to the transcriptional start site of the active chicken MYC promoter (Figure 2, upper graph). In contrast to the hypoacetylated status in chicken cells, the human MYC P2 gene promoter adopts an active configuration after transfer of human chromosome 8 into mouse B78 cells. In these cells, hyperacetylation of histones at the human MYC promoter is similar to that of the mouse MYC promoter, within a twofold range (Figure 2, lower graph). Hyperacetylation of histone H3 is detectable across an extended region of the wildtype human MYC gene, peaking at the upstream promoter region detected by primer set K (Figure 3) and at the transcription start sites of the P1 and P2 promoters (primer set N, Figure 3), analogous to our observations in human Jurkat and HL60 cell types [7]. With increasing distance from the transcription initiation sites, H3 acetylation decreases and is barely detectable within exon 3 and 2.3 kb upstream of exon 1 (primer sets R and L, respectively, in Figure 3). Primer sets that detect sequences further upstream of −2.3.kb and downstream of exon 3 did not detect any enrichment of H3 acetylation (data not shown). Together these data demonstrate that, in contrast to observations in chicken host cells, the human MYC allele on chromosome 8 adopts its normal chromatin configuration when transferred into murine host cells.

### The MYC insulator element does not affect hyperacetylation of the MYC chromatin domain

The addition of MINE to transgene constructs leads to an increase in transgene expression after integration into various genomic regions [7]. This result raises the question of whether MINE – in its native context - contributes to the maintenance of hyperacetylation in the MYC promoter region by inhibiting the silencing influence of flanking heterochromatin. To test this hypothesis, we used our panel of deletions within the upstream region of the MYC gene and defined the extent of histone acetylation. We performed ChIP analyses on both wildtype and mutant MYC alleles [wt, ΔLg, ΔCTCF-N, and ΔCTCF-NA] residing in murine B78 cells (Figure 3). Surprisingly, we found that the pattern of histone acetylation shows little if any change upon deletion of upstream regions. The promoter regions of all clones were hyperacetylated despite the deletion of sequences within the MINE and beyond. The ΔLg mutant allele - comprising an extensive 2.3 kb deletion surrounding the MINE – also generated a pattern comparable to the wild type allele (Figure 3). The level of histone hyperacetylation that is detected by primer set K remained unaffected, despite deletion of MINE and juxtaposition to sequences associated with hypoacetylated, “silent” chromatin. This suggests that the establishment of the hyperacetylated domain and the position of the boundary are mediated by promoter-proximal chromatin remodeling complexes that induce histone acetylation over a defined distance from the promoter.

In combination, these experiments demonstrate that targeted deletions of MINE and/or CTCF do not disrupt or increase the establishment of hyperacetylated chromatin at the MYC gene. Removal of the putative boundary by a deletion of upstream sequences as large as 2.3 kb (ΔLg) did not prevent or affect acetylation over the promoter, nor did deletion of any or both CTCF binding sites (CTCF-N and CTCF-NA influence the overall pattern of histone acetylation.

### CTCF is required for high-level MYC mRNA transcription

**Figure 4:**
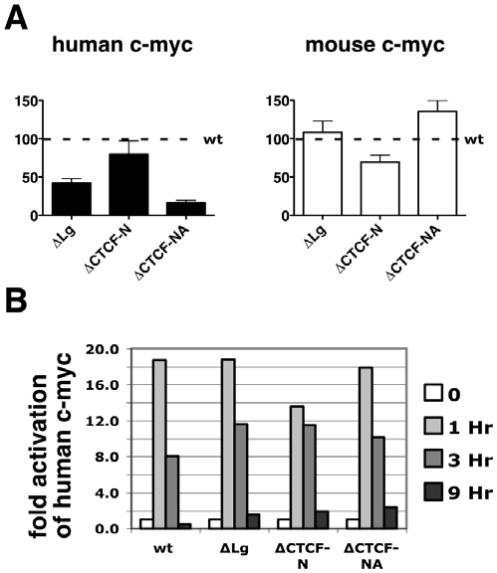
CTCF does not repress MYC expression and is required for normal levels of MYC mRNA. Real time RT-PCR analysis of the wildtype and recombinant MYC alleles residing on human chromosome 8 in B78 cells. (A) The steady-state level of human and mouse MYC RNA normalized to the endogenous mouse GAPDH mRNA. Left panel shows the relative expression of human MYC as a percentage of the expression detected in cells carrying the wildtype (wt) MYC allele. This analysis was performed multiple times for each B78 cell line containing a human MYC allele (wt, n = 7; ΔLg, n = 8; ΔCTCF-N, n = 9; ΔCTCF-NA, n = 6). Each clone was independently analyzed at least twice, and each PCR reaction was performed in duplicate. The bars indicate the mean value (±SEM) for all clones with the specific type of mutation. The right panel shows the expression of the endogenous murine MYC gene relative to the GAPDH gene. Mouse MYC gene expression is expressed as a percentage of the expression that is detected in cells carrying the human chromosome 8 with the wildtype allele. The expression in each of the mutant cells lines varies less than two-fold, indicating unaltered expression of the endogenous mouse MYC gene. (B) Expression of wildtype and mutant human MYC genes in starved (0 Hr) and serum-induced cells (1 Hr, 3 Hr, 9 Hr). RNA was harvested prior to serum induction (0 Hr), and 1 hour, 3 hours, and 9 hours post serum induction and analyzed by real-time RT-PCR. MYC mRNA levels were normalized to murine GAPDH mRNA levels. MYC expression in serum-starved cells was set to 1, and RNA levels at 1, 3 and 9 Hr are expressed as fold-induction over 0 Hr timepoint.

Previous studies using MYC promoter-driven reporter genes suggested that CTCF functions as a negative regulator of MYC expression. To address whether removal of the CTCF sites would also affect the level of MYC transcription in its native chromosomal site, we determined the relative abundance of human MYC mRNA by performing quantitative PCR in at least four independent fusion clones for each mutation made. MYC expression was normalized to expression of the endogenous murine GAPDH gene. Average transcript levels of the endogenous murine MYC gene varied less than twofold between the fusion clones carrying the wildtype and mutant human chromosomes, suggesting that the cell lines were kept under comparable physiologic conditions (Figure 4A). In contrast, comparison of the mean expression levels of the wildtype and mutated human MYC alleles differed up to 6-fold. The deletion of both CTCF sites in the mutant allele (CTCF-NA) significantly reduced the expression of MYC [16.3%±3.1 (mean±SEM, n = 6)] relative to the wildtype MYC allele (wt), whereas deletion of only the CTCF binding site N within MINE (ΔCTCF-N) did not effectively alter transcription (79.9%±17.2, n = 9). These results argue against a role for CTCF in transcriptional repression of MYC transcription. Removal of 2.3 kb of sequence encompassing the entire MINE also reduced MYC mRNA levels. It is important to note that this deletion removes multiple transcription factor binding sites, including the FUSE binding element known to be required for proper MYC expression [16]. Importantly, none of these deletions completely ablated MYC transcription. In concordance with previous reports [17], this argues against the presence of a ‘masterswitch’ element necessary for domain establishment and maintenance.

### CTCF is not required for transcriptional response to serum induction

MYC expression is highly responsive to growth factor signaling. While expression of MYC is virtually absent in serum-starved cells, its expression is induced 2–3 hours following addition of serum, and returns to a basal level after 9 hours. To determine whether the mutated human MYC alleles behave in a manner consistent with the normal MYC gene, we monitored induction of transcription at both the murine and human MYC genes in response to serum. B78 lines containing the wildtype and mutant human MYC alleles were starved for 24 hours, and the levels of human MYC mRNA were measured by quantitative PCR relative to the murine GAPDH gene before (0 hr) and at one, three and nine hours following addition of serum (Figure 4B). The serum-induced levels of MYC RNA (1 hr, 3 hr, 9 hr) were normalized to the RNA level before the addition of serum (0 hr). After addition of serum, MYC RNA levels in cells containing the wildtype allele increased 18-fold, and returned to a low steady-state level nine hours after addition of serum. Thus, the human MYC locus residing in mouse B78 cells is regulated by mitogenic signals similar to human MYC alleles in their normal context. Furthermore, removal of the CTCF binding sites at the MINE (ΔLg and ΔCTCF-N) and at the P2-promoter (ΔCTCF-NA) does not affect the ability of the human MYC allele to respond to serum induction (Figure 4B), suggesting that CTCF binding sites N and A are not necessary for the integration of multiple environmental signals during growth factor stimulation.

### CTCF deletion and epigenetic modifications at the human MYC allele

**Figure 5:**
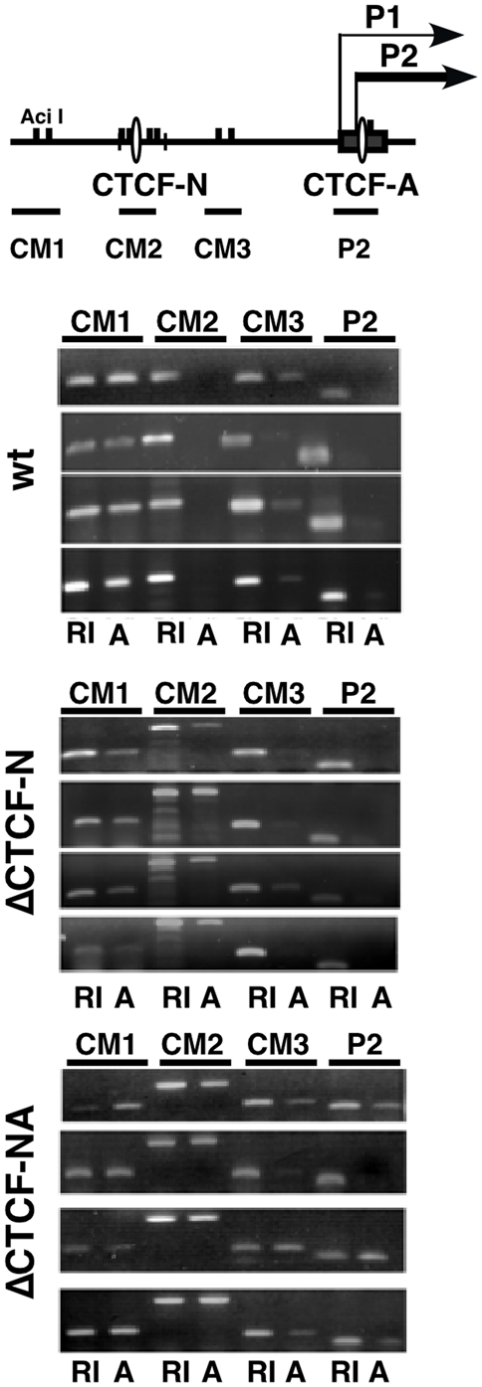
Deletion of CTCF binding sites protects the MYC promoter from DNA hypermethylation. Top, scheme of DNA methylation analysis in the MYC promoter region. *Aci*I restriction enzyme sites across the 2.5 kb human MYC 5′ region are indicated by filled squares. Positions of CTCF binding sites N and A, and of amplified regions using primer pairs CM1 to 3 [21] and P2 are indicated. DNA of 4 different clones for each allele (wt, ΔCTCF-N, ΔCTCF-NA) was digested with the methylation-sensitive restriction enzyme *Aci I* (A) or *EcoR I* (RI). Amplification of *EcoR I*-digested DNA serves as a positive control, and indicates the efficiency of PCR amplification. The level of amplification of *Aci I*-digested DNA indicates gain of DNA methylation in the tested region. While CM1 and CM3 regions show complete or partial methylation, CM2 and P2 regions are unmethylated in the wildtype human MYC allele. Deletion of CTCF sites N or N+A leads to DNA methylation.

The deletion of both CTCF sites N and A leads to reduced expression of the human MYC gene, suggesting that CTCF is required for maintaining high levels of transcriptional activity. Previous analyses of partially methylated viral reporter constructs demonstrated that methylation of genes within promoter-proximal regions and transcription start sites impair the efficiency of transcription initiation [18]. Intriguingly, CTCF has been reported to protect against de novo methylation of flanking sequences [19]–[21]. To test whether the decrease in MYC transcription at ΔCTCF-NA alleles in a cell population correlates with an increase in DNA methylation in the promoter region, we used a previously published protocol to measure the level of methylation within three promoter regions of the MYC gene [21]. In this assay, promoter regions of the human MYC gene are amplified with PCR using specific primer pairs CM1, CM2, and CM3 after treatment of genomic DNA with methylation-sensitive restriction enzymes (Figure 5). Successful PCR amplification indicates cytosine methylation; in contrast, a failure to amplify genomic DNA indicates unmethylated cytosine residues. Genomic DNA digested with Eco RI for which no restriction sites exist in the amplified regions was used as a reference. The methylation status of the CTCF site N within MINE (CM2) and its neighboring sites (CM1 and CM3) in the MYC locus were evaluated first. Similar to previous observations [21], CpG residues in the *Aci*I sites within CM1 were methylated in wildtype MYC alleles, as revealed by successful PCR amplification of this region. In contrast, the *Aci*I sites within genomic regions CM2 and P2 surrounding the CTCF site N and A, respectively, were hypomethylated (Figure 5). Partial methylation was observed in region CM3 in all cell lines, consistent with earlier reports [21]. When CTCF site N is deleted (ΔCTCF-N), DNA methylation in CM2 increases while the P2 promoter region remains largely unaffected. In contrast, deletion of both sites N and A increases DNA methylation in both regions, as indicated by successful amplification of these regions after digestion with the restriction enzyme *Aci* I. These results demonstrate that lack of CTCF binding at the MINE and the P2 results in a significant increase in DNA methylation at these sites. Together, our data suggest that CTCF binding at the MYC gene is required for high-level MYC expression. In the absence of CTCF binding, CpG residues at the P2 promoter region become methylated, reducing the ability to recruit transcription initiation complexes.

## Discussion

The zinc-finger protein CTCF is essential for the activity of most mammalian insulators and is thought to protect genes from unscheduled transcriptional activation by neighboring regulatory elements. Insulator activity is sometimes paired with barrier activity, which can prevent silencing of gene loci by inhibiting the spread of heterochromatin. In this study, we examined the role of CTCF binding sites within the MYC insulator element MINE in the context of its native position in human chromosome 8. In transgene experiments, MINE provides efficient insulator and barrier function when inserted into reporter gene constructs [7].

A global analysis of CTCF binding across the genomes of three different cell types identified a significant enrichment of CTCF sites at domain boundaries where histone modifications typical for transcriptionally active and inactive chromatin are juxtaposed [22]. Similarly, the CTCF site N of the human MYC gene is located 2.4 kb upstream of the MYC promoter at the intersection of transcriptionally active and inactive chromatin [7]. These findings suggest that CTCF may be involved in chromatin barrier function. However, deletion of CTCF sites N and A in the MYC promoter region has no effect on the level or extent of histone H3 acetylation in this domain (Figure 3). Consistent with this result, previous studies at the endogenous mouse β-globin locus have shown that deletion of the CTCF binding site within the 3′HS1 element has no effect on histone acetylation levels at the beta major gene [23]. In contrast, a reduction of histone acetylation upon deletion of the CTCF binding motif has been observed in episomal reporter genes that contain a cHS4 insulator element between enhancer and promoter [24]. While the exact reason for this discrepancy remains unknown, these observations emphasize that CTCF's function is strongly context-dependent. Thus, in order to fully understand the impact of CTCF on gene expression functional studies should ideally be performed in the normal genomic locale of a given gene.

Our analysis of CTCF activity at the human MYC gene in the context of human chromosome 8 surprisingly indicated a requirement for CTCF for high-level expression of the MYC gene. While the removal of a single CTCF site showed little influence on MYC expression, deletion of both sites N and A led to a decrease in MYC expression. This result suggests that deletion of CTCF binding at site A is important for full level expression of the human MYC gene. However, it is important to note that the 59 bp deletion downstream of the P2 transcriptional start site may have affected binding of neighboring regulatory factors or their relative spacing to each other. Although we are not aware of reports describing transcription factor binding within the deleted region of our ΔCTCF-NA mutant, we cannot rule out the possibility that the deletion of CTCF has no effect at all on transcriptional activity and that observed effects are due to displacement of factors other than CTCF. Nevertheless, our results are inconsistent with the previously reported role for CTCF as a repressor of MYC activity that has been observed with transiently transfected MYC reporter constructs in the presence of high levels of CTCF [6]. The discrepancy between these two results is likely due to the sensitivity of CTCF to the sequence context used in different experimental approaches. The essential role for CTCF in nuclear organization and inter/intrachromosomal interactions might affect the localization of transfected templates and chromosomal regions in different nuclear compartments. Thus, the previously identified multi-functional activities of CTCF in transcriptional activation and repression may be a consequence of the tethering of reporter genes to different nuclear loci.

The decrease of MYC transcriptional activity upon deletion of CTCF binding sites correlates with an increase in the frequency of methylation at CpG residues, consistent with earlier reports of a role for CTCF in the establishment of DNA methylation free domains [2], [25]–[27]. The deletion of CTCF binding sites N and A in the mutant ΔCTCF-NA allele leads to a significant decrease in transcriptional activity (less than 20% of the wild type human MYC allele) and correlates with an increase in methylation at the CM2 and P2 sites. Despite an increase in DNA methylation, hyperacetylated histones are still detectable in these cell lines. Although examples of transcriptionally active genes with coexisting histone acetylation and local DNA methylation have been reported [28], this observation may also reflect heterogeneous MYC expression in distinct subsets of cells in our mutant lines. Additional studies using positive selection markers will be required to distinguish between these possibilities.

The increase of local DNA methylation upon deletion of CTCF sites is consistent with previous reports describing a role for CTCF in maintaining DNA methylation-free domains [20], [21], [25], [27], [29]. While the exact role of CTCF in protecting CpG residues from methylation is unknown, the previously reported CTCF-mediated activation of poly(ADP-ribose)-polymerase (PARP1) provides an intriguing link to its role in inhibition of DNA methylation (reviewed in [30], [31]).

In summary, our results demonstrate a role for CTCF in the maintenance of MYC gene expression. While our data are inconsistent with a role for CTCF in transcriptional suppression of MYC, the controversial findings of the diverse functions of CTCF may be due to its role in nuclear organization and long-range interactions. CTCF may mediate transcriptional activation, suppression, and insulation through its ability to form inter- and intrachromosomal chromatin loops. The previous discovery of co-localization of CTCF and cohesin across the mammalian genome may provide a basis for the functional interplay between nuclear organization and regulation of gene expression [32]–[35].

## Materials and Methods

### Cell lines and culture conditions

DT40[h8n] is a derivative of the chicken B cell-line DT40, and harbors a single intact human chromosome 8 containing a neomycin resistance gene [15]. Cells were maintained in Dulbeccos's modified Eagle's media (DME) supplemented with 10% fetal calf serum/1% chicken serum, 10% tryptone phosphate, and selection media (1 mg/ml G418). The murine melanoma cell line B78 was propagated in DME supplemented with 5% Fetalplex (Geminibiosources, Atlanta). To select for the presence of *neo^r^*-tagged human chromosome 8 in hybrid cell-lines [B78(h8n-wt), B78(ΔLg), B78(ΔCTCF-N), B78(ΔCTCF-NA)], cells were cultured in 0.5 mg/ml G418.

### DNA transfections

1.5–3.0 pmol linearized plasmid was introduced into cells by electroporation as described [15]. Electroporated cells were subsequently selected with 2 mg/ml hygromycin or with 1.5 mg/ml L-histidinol and 1 mg/ml G418 for 14–20 days. Cell lines with properly integrated hygromycin*^r^* selection cassettes were transiently transfected with CRE-recombinase/GFP-expression plasmid introduced by electroporation. After establishing cell lines from single GFP positive cells, the successful excision of the selection cassettes by CRE was analyzed by selection of cells in hygromycin or histidinol. Correct homologous recombinant events were identified by PCR and Southern hybridization.

### Transfer of human chromosome 8

To transfer human chromosomes (containing either wt, ΔLg, ΔCTCF-N or ΔCTCF-NA MYC alleles) from DT40 into B78 cells, we used a suspension-monolayer fusion protocol [36] to generate whole-cell fusions between donor cells (e.g. DT40[h8n] (*neo* ^R^, ouabain^S^) and recipient B78 cells (*neo* ^S^, oubain^R^)). Hybrid clones were selected in 0.5 mg/ml G418 and 20 µM ouabain. The intactness of the human chromosomes residing in B78 hybrids was assessed by sequence-tagged site (STS) marker PCR analysis (STS-PCR primer pairs for human chromosome 8 are available upon request) and fluorescent *in situ* hybridization (FISH) karyotyping using normal human fibroblast genomic DNA as a probe. Human DNA was labeled with biotin-14-dATP and detected with FITC-avidin as described [37]. Slides were counterstained with DAPI and examined with an epifluorescence microscope. Clones that carried an intact copy of human chromosome 8 in a non-rearranged form were used for further analysis of human MYC expression.

### Chromatin immunoprecipitation (ChIP) assays

ChIP assays were performed essentially as described previously [7]. Chromatin was cross-linked in the presence of 1% formaldehyde for 3 min at room temperature. After the addition of glycine to a 0.125 M final concentration, cells were washed in ice-cold phosphate-buffered saline containing protease inhibitors, pelleted, resuspended in sodium dodecyl sulfate (SDS) lysis buffer (1% SDS, 10 mM EDTA, 50 mM Tris-HCl (pH 8.1), 1× protease inhibitor cocktail, and 10 mM butyrate), and sonicated 15 times for 30 s each at setting 6.5 in a Branson sonicator with a microtip. The DNA length ranged from 200 bp to 800 bp. After centrifugation to remove cell debris, the whole-cell extract was diluted 10-fold with ChIP dilution buffer (1% Triton X-100, 2 mM EDTA, 20 mM Tris-HCl [pH 8.1], 150 mM NaCl, 1× protease inhibitor cocktail and 10 mM butyrate). This chromatin solution was cleared before immunoprecipitation using protein A agarose slurry and normal rabbit serum to reduce nonspecific background. Anti-acetylated histone H3 or anti-CTCF antibody (06-599 and 06-917, Upstate Biotechnology) was added, and the mixture was incubated overnight at 4°C. Immune complexes were collected by addition of 25 µL protein A agarose slurry that had been preincubated with bovine serum albumin and salmon sperm DNA. After incubation at 4°C for 2 hours, the agarose beads were washed several times as described [38]. DNA recovered from immunoprecipitations was analyzed by duplex PCR using MYC-specific primers and reference primers (*β-globin*) (primer sequences available upon request) in the presence of [32P]dCTP. The fold enrichment in each immunoprecipitation reaction was determined by calculating the ratio of the signal obtained with MYC primers to that of the reference primers.

### Quantitative estimation of mRNA by Real Time PCR analysis

Synthesis of cDNA was carried out according to the manufacturers instructions (Qiagen) using 1 ug of total RNA. Reverse transcription was carried out at 37°C for one hour. Real Time PCR reactions were performed in 20 µl volume on an ABI Prism-7000 (Applied Biosystems), in accordance with the recommended protocol. TaqMan®-based (Applied Biosystems) experimental protocols were used to determine the expression of murine GAPDH (Mm99999915_g1), murine MYC (Mm00487803_m1), and human MYC (Hs00153408_m1). The TaqMan Universal PCR Master Mix™ protocol was used with each probe according to the manufacture's instructions. Each PCR was carried out in duplicate to control for PCR variation. The relative abundance of each target was calculated using the cycle threshold (Ct) values, using the formula 2^−CT^. The relative expression of each target was normalized to murine GAPDH, using the formula x = 2^−ΔΔCt^, where x equals the fold difference in the amount of RNA between the wild type and the deleted mutation clones (ΔΔCt = ΔCt_wt_ − ΔCt_deletion mutant_ and ΔCt_wt_ = Ct *_c-myc_*− Ct_GAPDH_). Each calculation was normalized to murine GAPDH and then expressed as a percentage of the wild type calculation.

### DNA Methylation Analysis

Genomic DNA was extracted according to manufacturer's instructions and resuspended in TE buffer (DNAeasy™, Qiagen). For each sample, 1 µg DNA was digested with *Hpa*II, *Aci*I, or *EcoR*I. 100 ng digested DNA was PCR amplified using site-specific primers (CM1-4) as described [21]. In addition, three primer sets were used to analyze the MINE CTCF region (F73/R266): GCCATTACCGGTTCTCCATA/CAGGCGGTTCCTTAAACAA; CM(OuS)/R596: CCCGCGTTTGCGGCAAA/CAGCTCAGCGTTCAAGTGTT; and CM(InS)/R596: GGTCCCTCGAAGAGGTTCAC/CAGCTCAGCGTTCAAGTGTT) and one set the P2-CTCF site (F2444/R2611: GGGATCGCGCTGAGTATAAA/CCTATTCGCTCCGGATCTC). At least three different independent clones were studied for each mutation.
